# Gefitinib in treatment of metastatic non-small cell lung cancer (NSCLC) with mutated epidermal growth factor receptor (EGFR)

**DOI:** 10.1186/2051-1426-2-S3-P187

**Published:** 2014-11-06

**Authors:** Charu Singh

**Affiliations:** 1 S.M.S Medical College and Hospital, JAIPUR, India

## Aim

To evaluate the efficacy, toxicity, overall survival and response of Gefitinib in previously untreated patients of metastatic NSCLC with EGFR mutation.

## Methods

60 patients with metastatic, non-small-cell lung cancer and EGFR mutations who had not previously received chemotherapy were randomly assigned to receive Gefitinib 250 mg orally daily or carboplatin -paclitaxel. The primary end point was progression-free survival; secondary end points included overall survival, response rate, and toxic effects.

## Results

The progression free survival was significanly longer in Gefitinb group than in standard chemotherapy group. The Gefitinib group had significantly longer median progression free survival (10 months versus 5 months) as well as higher response rates (70% versus 30%). The median overall survival was 30 months in Gefitinib group and 24 months in chemotherapy group. The most common adverse events in Gefitinib group were rash and elevated aminotransferase levels.

## Conclusions

First-line Gefitinib for patients with advanced non-small-cell lung cancer who were selected on the basis of EGFR mutations improved progression-free survival, with acceptable toxicity, as compared with standard chemotherapy.

